# Inter-study reproducibility of cardiovascular magnetic resonance-derived hemodynamic force assessments

**DOI:** 10.1038/s41598-023-50405-9

**Published:** 2024-01-05

**Authors:** Torben Lange, Sören J. Backhaus, Alexander Schulz, Ruben Evertz, Patrick Schneider, Johannes T. Kowallick, Gerd Hasenfuß, Sebastian Kelle, Andreas Schuster

**Affiliations:** 1 Department of Cardiology and Pneumology, University Medical Center Göttingen, Georg-August University, Robert-Koch-Str. 40, 37099 Göttingen, Germany; 2 German Center for Cardiovascular Research (DZHK), Partner Site Göttingen, Göttingen, Germany; 3 School of Biomedical Engineering and Imaging Sciences, King’s College London, London, UK; 4 Institute for Diagnostic and Interventional Radiology, Georg-August University, University Medical Center Göttingen, Göttingen, Germany; 5 Department of Internal Medicine/Cardiology, Charité Campus Virchow Clinic, Berlin, Germany; 6 German Centre for Cardiovascular Research (DZHK), Partner Site Berlin, Berlin, Germany

**Keywords:** Cardiology, Diagnostic markers

## Abstract

Cardiovascular magnetic resonance (CMR)-derived hemodynamic force (HDF) analyses have been introduced recently enabling more in-depth cardiac function evaluation. Inter-study reproducibility is important for a widespread clinical use but has not been quantified for this novel CMR post-processing tool yet. Serial CMR imaging was performed in 11 healthy participants in a median interval of 63 days (range 49–87). HDF assessment included left ventricular (LV) longitudinal, systolic peak and impulse, systolic/diastolic transition, diastolic deceleration as well as atrial thrust acceleration forces. Inter-study reproducibility and study sample sizes required to demonstrate 10%, 15% or 20% relative changes of HDF measurements were calculated. In addition, intra- and inter-observer analyses were performed. Intra- and inter-observer reproducibility was excellent for all HDF parameters according to intraclass correlation coefficient (ICC) values (> 0.80 for all). Inter-study reproducibility of all HDF parameters was excellent (ICC ≥ 0.80 for all) with systolic parameters showing lower coeffients of variation (CoV) than diastolic measurements (CoV 15.2% for systolic impulse vs. CoV 30.9% for atrial thrust). Calculated sample sizes to detect relative changes ranged from n = 12 for the detection of a 20% relative change in systolic impulse to n = 200 for the detection of 10% relative change in atrial thrust. Overall inter-study reproducibility of CMR-derived HDF assessments was sufficient with systolic HDF measurements showing lower inter-study variation than diastolic HDF analyses.

## Introduction

Cardiovascular magnetic resonance (CMR) imaging has been established as a key imaging modality for comprehensive myocardial function assessment in clinical cardiology over the past decades^1,2^. Within the scope of cardiac deformation analyses, especially CMR-derived myocardial strain assessments have been proven to possess accurate diagnostic capabilities and to provide substantial prognostic information^3–6^. Despite several clinically approved and widely used imaging biomarkers, efforts and new developments in non-invasive imaging strive for identifying even more sensitive and precise parameters of subclinical failing cardiac performance^7^. In this context, hemodynamic force (HDF) assessment provides novel and promising imaging parameters representing the force exchange between ventricular blood and surrounding myocardium but required knowledge of intracardiac velocity fields measured by CMR-based 4D flow tools up to now. Recently, CMR-based HDF analyses derived from routinely acquired CMR cine images have been introduced allowing non-invasive estimation of intracardiac pressure gradients solely based on LV geometry, endocardial tissue motion as well as aortic and mitral valve orifice areas^8,9^. Based on the assumption that HDF is mainly influenced by flow properties following myocardial deformation and the exchange of momentum across the mitral and aortic orifices, this novel approach estimates main features of HDF vectors from endocardial tissue dynamics. Of note, this novel method has been proven to possess high accuracy when validated against conventional 4D flow-based assessments^10^.

Importantly, HDF analyses were recently demonstrated to improve detection of early cardiac disease and myocardial dysfunction when volumetric and deformation cardiac measures are still intact^9^. There is even evidence to suggest that this technology identifies subtle systolic alterations in heart failure with preserved ejection fraction (HFpEF) that cannot be picked up with alternative technology^11,12^. However, for an unrestricted clinical use and in order to provide robust and valid measurements, image post-processing applications must be sufficiently reproducible^13^.

Consequently, the aim of this study was to evaluate the inter-study reproducibility of CMR cine image-based HDF assessments and to define the current potential and limitations of this novel deformation imaging tool.

## Methods

### Study population

The study population included 11 healthy participants (6 males and 5 females) with a mean age of 46 years (± 23 years). Study participants underwent CMR twice at a median interval of 63 days (IQR 49–87 days) using a standardized CMR scan protocol^14,15^. To avoid a recollection bias of the involved CMR staff a minimum time gap of 6 weeks between both scans was determined. Particular emphasis was laid on performing image acquisitions at the same anatomical levels of the heart. Furthermore, new onset of a cardiac disease was excluded before the second CMR scan. All subjects had stable sinus rhythm during CMR acquisition. The study was approved by the Ethics Committee of the Charité-University Medicine Berlin (registration: EA4/112/16; German Clinical Trials Registry, DRKS, DRKS00015615). All subjects gave written informed consent before participation and the study was conducted according to the principles of the Helsinki Declaration.

### Cardiovascular magnetic resonance imaging

All subjects underwent CMR imaging using a standardized scanning protocol on a 1.5 Tesla scanner (Achieva, Philips Healthcare, Best, The Netherlands) equipped with a cardiac, five-element phased array coil. Conventional retrospectively ECG-gated balanced steady state free precession (bSSFP) cine sequences were acquired during breath-hold for the assessment of myocardial function. 2-dimensional cine sequences included 2-, 3- and 4- chamber views (CV) in long axis (LAX) orientation as well as short axis (SAX) stacks covering the entire left ventricle. Imaging parameters were as follows: 50 frames/ cardiac cycle*,* repetition time (TR) = 3.3 ms, echo time (TE) = 1.6 ms, flip angle = 60° and acquisition voxel size = 1.8 × 1.7 × 8.0 mm^3^. The same imaging parameters were applied for baseline and repeated CMR scans.

**Figure 1:**
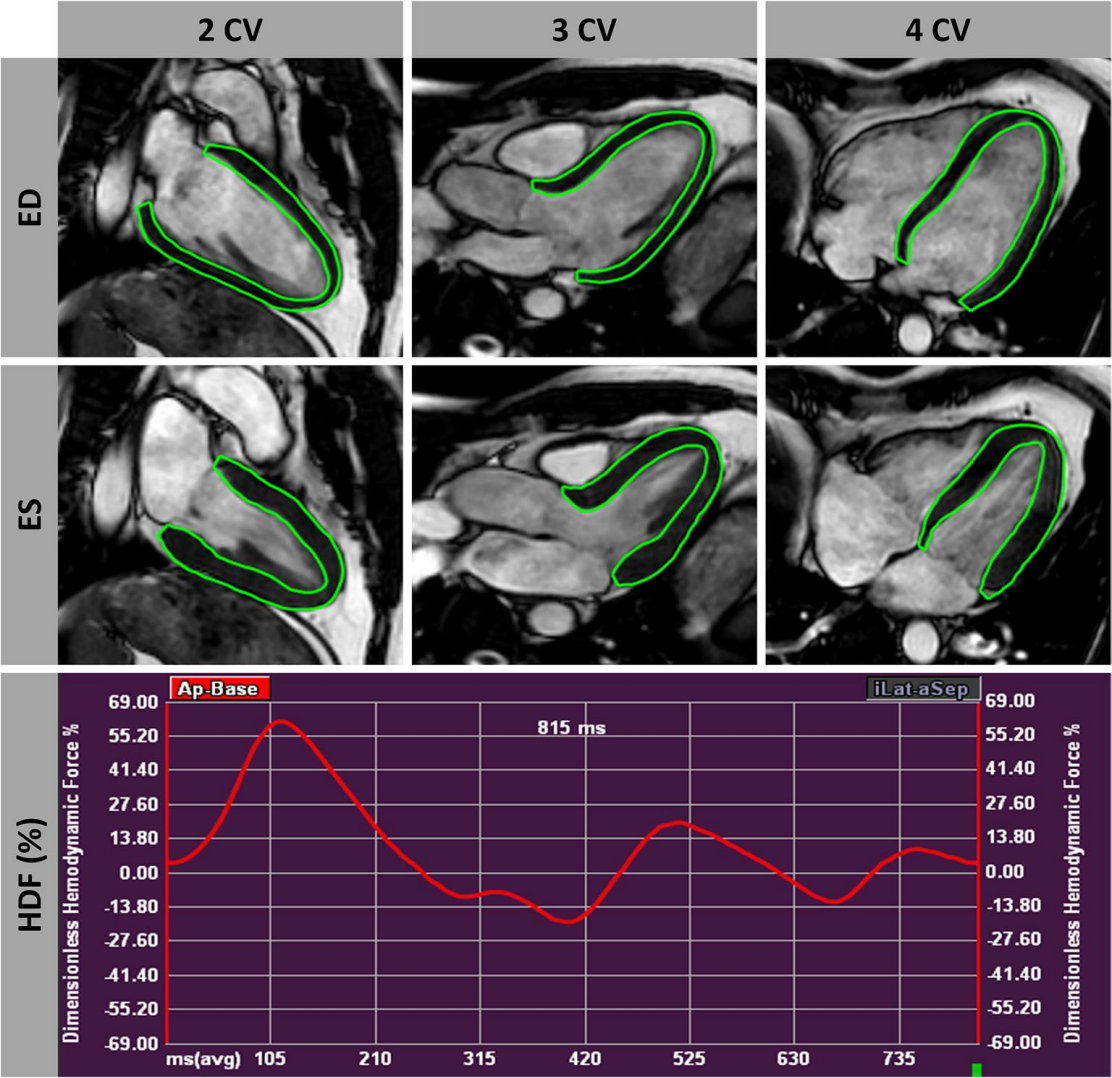
Myocardial delineation for hemodynamic force analysis. Delineated epi- and endocardial contours in long axis 2-/3-/4-chamber view (CV) orientations during enddiastole (ED) and endsystole (ES) with a corresponding hemodynamic force (HDF) curve.

Dedicated commercially available software was used for CMR image post-processing (QStrain® and HDF module, Medical Imaging Systems, Leiden, Netherlands). Movements from deformation imaging of 2-,3- and 4-CV were used for apical-basal LV HDF calculation after initial feature-tracking (FT)^16^ and measurements of mitral and aortic valve width (Fig. 1)*.* HDF evaluations report an integral of LV pressure gradients. To correct for different LV sizes and to facilitate comparisons between participants HDF evaluations are normalized to LV volume and blood specific weight and presented in percentage of gravity acceleration. Detailed HDF calculations and more mathematical features of the method transforming endocardial dynamics into flow forces have been described elsewhere^17,18^.

In brief, the general relationship between pressure gradient and velocity field can be derived from the Navier‐Stokes equation. The original formula required blood velocity (e.g., measured by 4D flow) but could be adapted so that this variable can be computed from an integral over the LV endocardial boundary allowing evaluation HDF from the knowledge of the moving ventricular geometry and the valve orifice. The HDF calculations in this study comprised the longitudinal force over an entire cardiac cycle with the direction of HDF pointing from higher towards lower pressure areas. Briefly explained, during the beginning of systole, apical-basal pressure gradients cause a positive deflection of the HDF curve resulting in blood ejection from the LV. After reaching the peak of systolic impulse, tension of the LV contraction decreases and the apical-basal gradient changes into a descending but still positive HDF curve.

**Figure 2:**
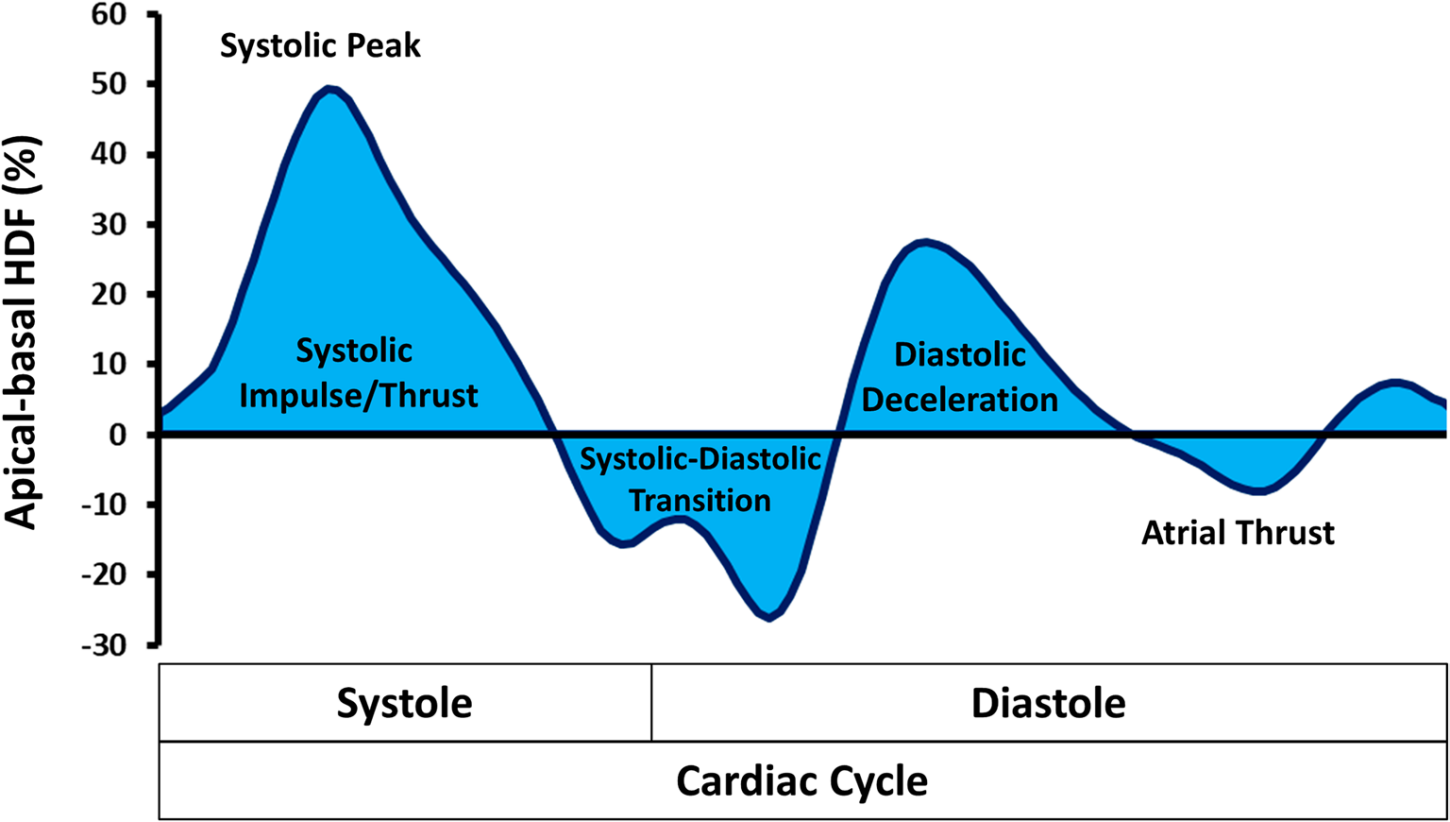
Left ventricular hemodynamic force analysis in apical-basal direction. Illustration of an exemplary hemodynamic force (HDF) curve for the left ventricular apical-basal motion over a whole cardiac cycle. Measured quantitative HDF metrics correspond to the area under the curve of each HDF phase.

Subsequently, with decelerating ventricular flow and aortic pressure surpassing the ventricular pressure, the apical-basal gradient reverses, which is depicted by the first half of the negative systolic-diastolic transition curve. After the aortic valve has closed, diastole begins and caused by relaxation and recoil of the LV myocardium, an early diastolic suction occurs generating a basal–apical gradient, that is represented by the second half of the negative systolic-diastolic transition curve. Subsequent diastolic deceleration is characterized by passive LV filling and upward movement of the mitral plane. In this phase, first LV pressure increases from apex to base and makes HDF grow in the positive ascending phase. By exceeding the atrial pressure LV filling decelerates and reduced passage of blood from the atrium to the LV progressively equilibrates the pressures in both chambers resulting in a positive but descending phase on the HDF curve. Atrial thrust reflects late diastolic filling due to atrial contraction, that causes a relative gradient from apex to base, resulting in negative HDF vectors. Finally, as the blood accumulates in the LV chamber, the gradient reverses again and the HDF vector becomes positive before systolic impulse begins^18^ (Fig. 2).

The mean longitudinal force amplitude over the entire cardiac cycle is expressed as a dimensionless root mean square (RMS) considering both positive and negative values. The peak of the systolic impulse curve was defined as systolic peak HDF. All HDF parameters were calculated from the area under the curve (AUC) normalised to the respective time interval and are reported as average values based on three independently repeated measurements^19^*.* In addition to the HDF measurements LV global longitudinal strain (GLS) values were assessed in LAX 2-, 3- and 4-CV cine images^20^.

Post-processing was performed by an experienced observer. The same observer repeated feature-tracking-based myocardial border delineation as well as measurements of mitral and aortic valve width on the same data-set to assess intra-observer variability. Similarly, a second observer performed feature-tracking in LAX orientations and measurement of valve widths for the calculation of inter-observer reproducibility.

Volumetric analyses were performed in LV SAX orientations comprising LV enddiastolic/-systolic and stroke volumes (EDV/ESV/SV) as well as LV ejection fraction (EF) and mass.

### Statistical analyses

Statistical analyses were performed using SPSS version 28.0 (IBM, Armonk, New York, USA) and Microsoft Excel. All p-values are provided 2-tailed and an alpha level < 0.05 was considered statistically significant. Continuous parameters are reported as mean along corresponding standard deviations (SD). For dependent continuous parameters changes from Exam 1 to 2 were evaluated applying the Wilcoxon signed-rank test.

Inter-study and inter-observer variability was analysed using intra-class correlation coefficients (ICC) based on absolute agreement (excellent for ICC > 0.74, good between 0.60 and 0.74, fair between 0.4 and 0.59 and poor below 0.4)^13^, coefficient of variation (CoV, SD of mean difference divided by the mean (SD [mean difference])/mean) and Bland–Altman plots (mean difference between measurements with 95% confidence interval [CI])^21^.

Study samples required to show a respective 10%, 15% and 20% relative change in HDF measurements with a power of 90% and an α error of 0.05 were calculated as follows^22,23^:

$${\text{n}} = {\text{f}}\;\left( {\alpha ,{\text{ P}}} \right)*\sigma^{2} *\left( {2/\delta^{2} } \right)$$

where n is the sample size, α the significance level, P the study power required and f the value of the factor for different values of α and P (f = 10.5 for α = 0.05 and p = 0.90), with σ the inter-study SD and δ the desired difference to be detected^23^.

## Results

### Cardiovascular magnetic resonance imaging

**Figure 3:**
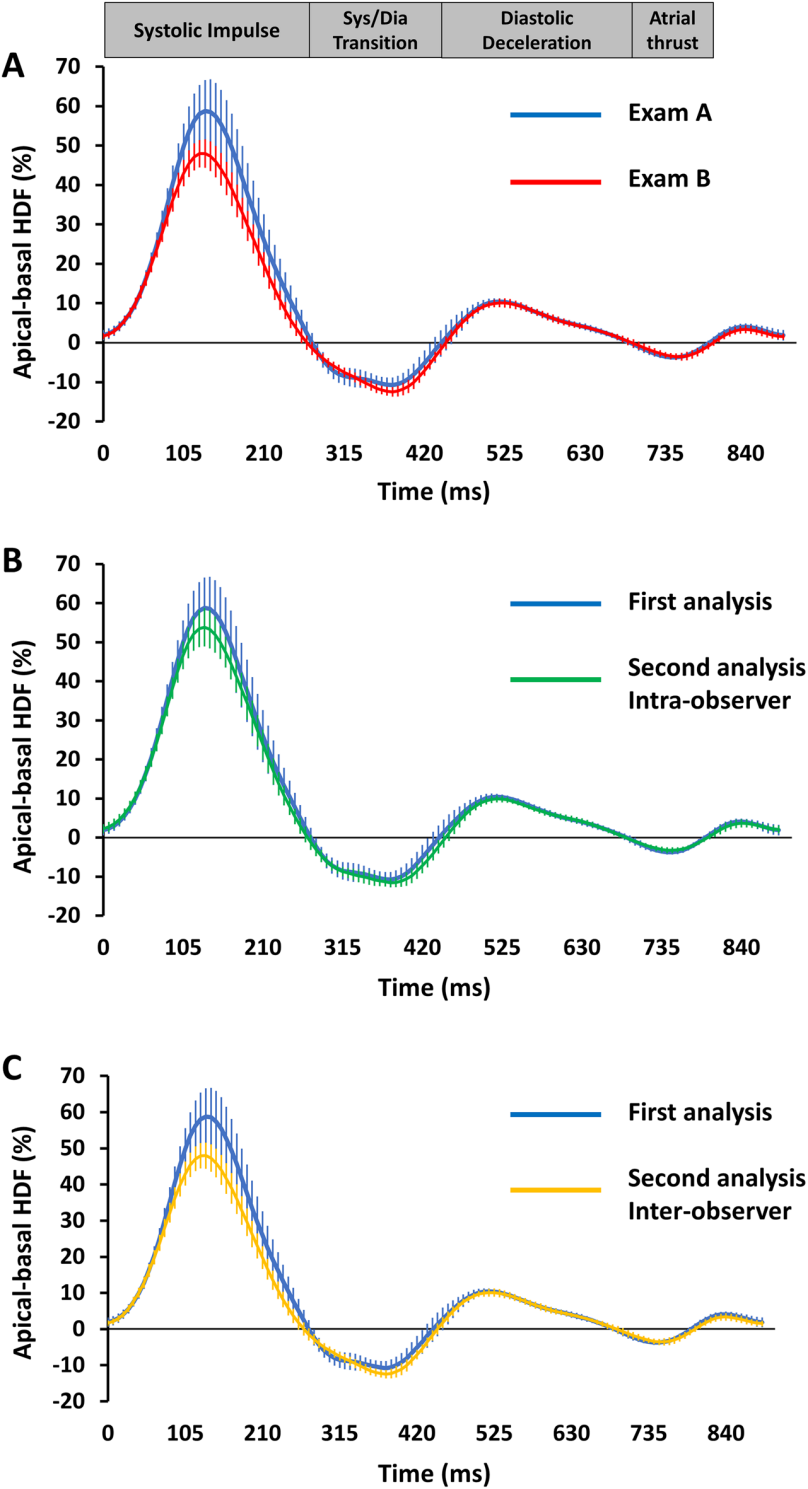
Hemodynamic force analyses profiles. Hemodynamic force (HDF) analyses profiles are displayed for both exams. The blue curve (first exam) and red curve (second exam) represent the respective average of the whole study group with the respective 95% confidence interval of the measurements (**A**). HDF analyses profiles for intra- (**B**) and inter-observer (**C**) measurements based on the first exam each representing the averages of the whole study group with the respective 95% confidence intervals (please see colour codes).

Results of CMR analyses of both exams are summarized in Table 1. HDF profiles of all repetitions are presented in Fig. 3. Neither LV volumes nor LV GLS differed significantly between both exams, only LV mass showed a significant difference (91.3 vs. 88.2 g/m^2^; p = 0.02). Amongst HDF parameters, there were significant differences between both exams for the values of LV longitudinal force (p = 0.03), systolic peak (p = 0.02) and systolic impulse (p = 0.04), while the other HDF measurements did not differ significantly.

**Table 1:** Cardiovascular magnetic resonance image analyses.

Variable	Exam 1	Exam 2	p-value
Cardiovascular magnetic resonance function			
LV EF (%)	61.0 (57.0–62.8)	59.8 (56.6–62.2)	0.37
LV EDV (ml/m^2^ BSA)	151.4 (133.6–175.9)	145.2 (136.6–173.7)	0.1
LV ESV (ml/m^2^ BSA)	58.7 (53.5–72.9)	58.2 (54.9–69.3)	0.93
LV SV (ml/m^2^ BSA)	93.9 (76.8–105.8)	91.5 (79.6–102.8)	0.06
LV Mass (g/m^2^ BSA)	86.1 (73.5–106.7)	83.0 (71.0–106.1)	**0.02**
LV GLS (%)	− 20.9 (− 19.4 to − 22.1)	− 21.1 (− 18.8 to − 22.1)	0.93
Cardiovascular magnetic resonance hemodynamic force			
LV longitudinal force (RMS) (%)	21.7 (18.1 − 28.1)	19.1 (17.6–23.7)	**0.03**
Systolic peak (%)	60.5 (55.1–89.0)	52.3 (51.4–69.9)	**0.02**
Systolic impulse (%)	34.0 (31.9–48.0)	31.8 (29.6–40.9)	**0.04**
LV systolic/diastolic transition (%)	− 11.1 (− 10.2 to − 12.4)	− 10.7 (− 9.9 to − 13.6)	0.4
Diastolic deceleration (%)	8.4 (4.3–12.1)	7.2 (5.0–9.1)	0.13
Atrial thrust (%)	− 4.1 (− 1.8 to − 4.5)	− 2.5 (− 2.0 to − 4.5)	0.16
Diastolic deceleration/atrial thrust (%)	2.3 (1.4–3.3)	2.1 (1.7–4.4)	0.79

Volumes are given in ml/m^2^ body surface area (BSA), mass in g/m^2^ BSA, strain and HDF values in %. Independent continuous parameters are presented as median with interquartile range and were compared by using the Wilcoxon signed-rank test. Bold p-values indicate statistical significance.

*LV* left ventricular, *EDV* enddiastolic volume, *ESV* endsystolic volume, *SV* stroke volume, *EF* ejection fraction, *GLS* global longitudinal strain, *RMS* root mean square.

### Inter-study and observer reproducibility

**Figure 4:**
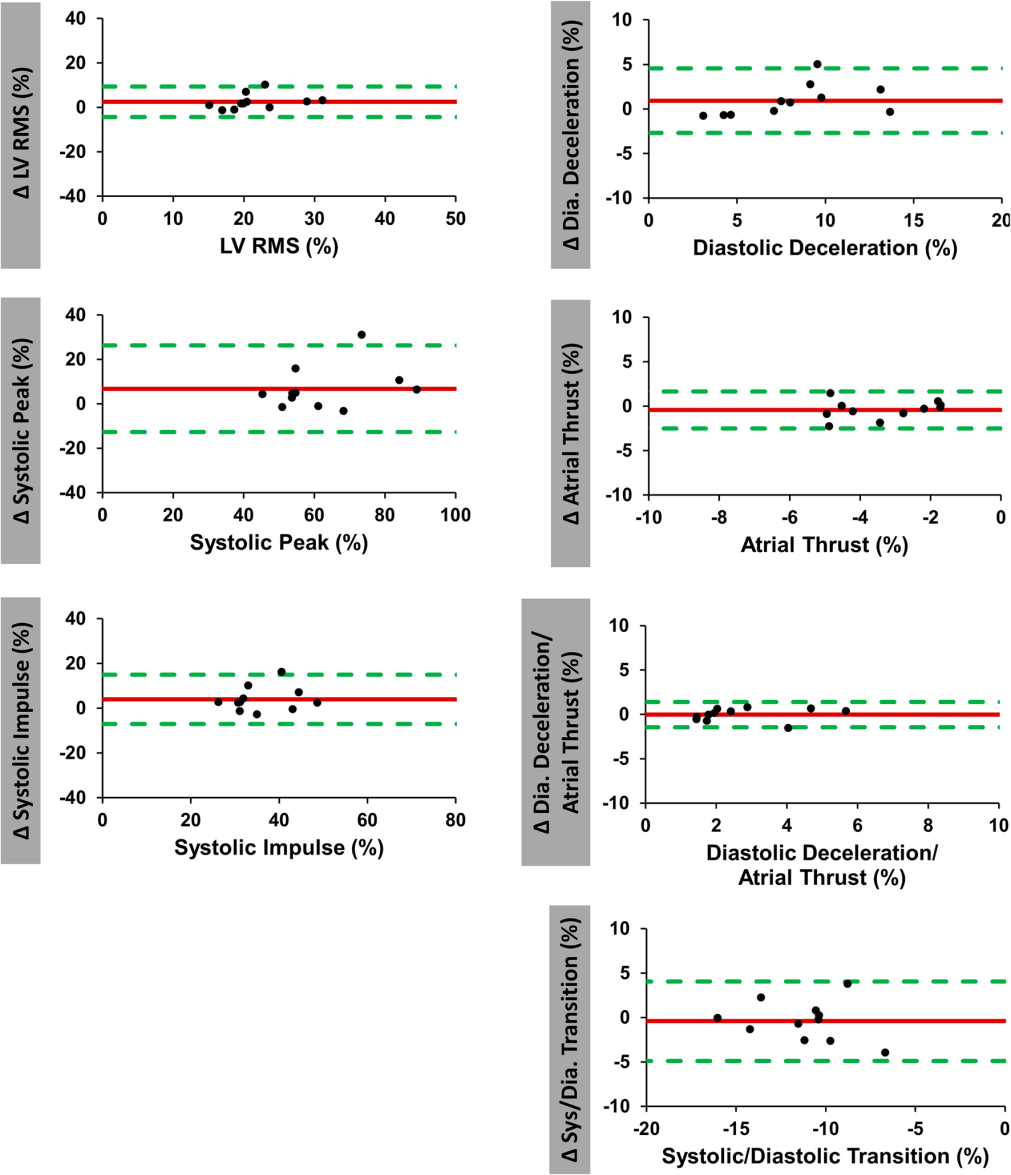
Inter-study agreement of hemodynamic force analyses. Bland Altman plots with limits of agreement (95% confidence intervals) showing inter-study reproducibility of hemodynamic force (HDF) analyses. Delta values (Δ) displaying the difference for inter-study measurements. *RMS* root mean square, *LV* left ventricular.

Results of inter-study reproducibility are reported in Table 2 and Fig. 4. Overall reproducibility was excellent for all HDF values according to ICC (ICC ≥ 0.80 for all). Atrial thrust (CoV 30.9%), the ratio of diastolic deceleration/atrial thrust (CoV 25.9%) and diastolic deceleration (CoV 22.2%) showed notably higher inter-study variability than systolic HDF measures (systolic impulse: CoV 15.2% and systolic peak: 15.6%).

**Table 2:** Inter-study reproducibility.

Variable	Mean difference ± SD	Coefficient of variation (%)	ICC (95% CI)
LV longitudinal force (RMS) (%)	2.5 (± 3.4)	15.8	0.83 (0.3–0.96)
Systolic peak (%)	6.8 (± 9.7)	15.6	0.84 (0.37–0.96)
Systolic impulse (%)	3.9 (± 5.5)	15.2	0.8 (0.2–0.95)
LV systolic/diastolic transition (%)	0.4 (± 2.2)	21.5	0.83 (0.36–0.95)
Diastolic deceleration (%)	0.9 (± 1.8)	22.2	0.92 (0.7–0.98)
Atrial thrust (%)	0.4 (± 1.0)	30.9	0.85 (0.46–0.96)
Diastolic deceleration/atrial thrust (%)	0.04 (± 0.7)	25.9	0.94 (0.79–0.99)
LV global longitudinal strain (GLS) (%)	0.1 (± 2.1)	10.3	0.86 (0.45–0.96)
LV ejection fraction (LVEF) (%)	0.6 (± 2.8)	3.1	0.91 (0.67–0.97)

*CI* confidence interval, *ICC* intraclass correlation coefficient, *LV* left ventricular, *RMS* root mean square, *SD* standard deviation, *GLS* global longitudinal strain, *LVEF* LV ejection fraction.

**Figure 5:**
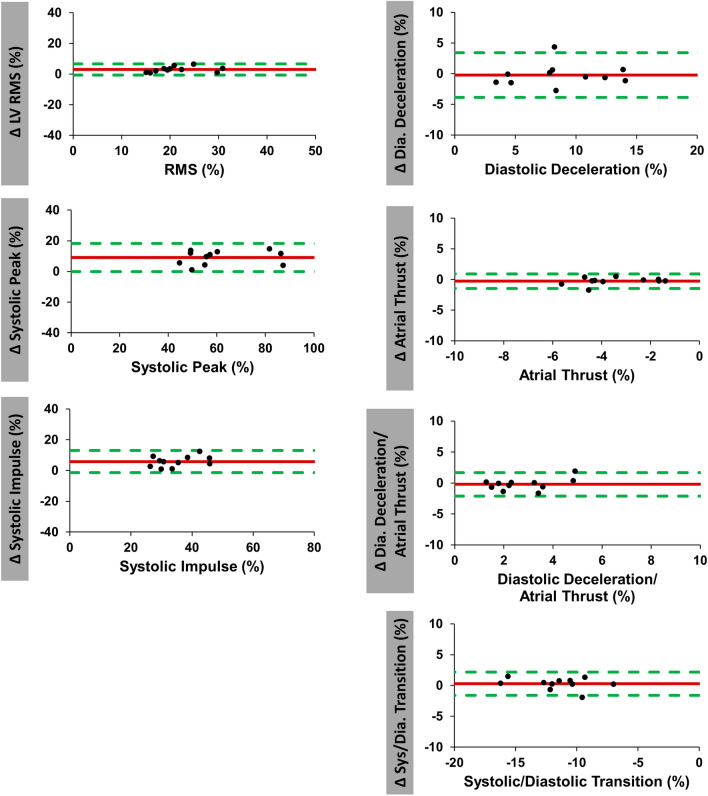
Intra-observer agreement of hemodynamic force analyses. Bland Altman plots with limits of agreement (95% confidence intervals) showing intra-observer reproducibility of hemodynamic force (HDF) analyses. Delta values (Δ) displaying the difference for intra-observer measurements. *RMS* root mean square, *LV* left ventricular.

**Figure 6:**
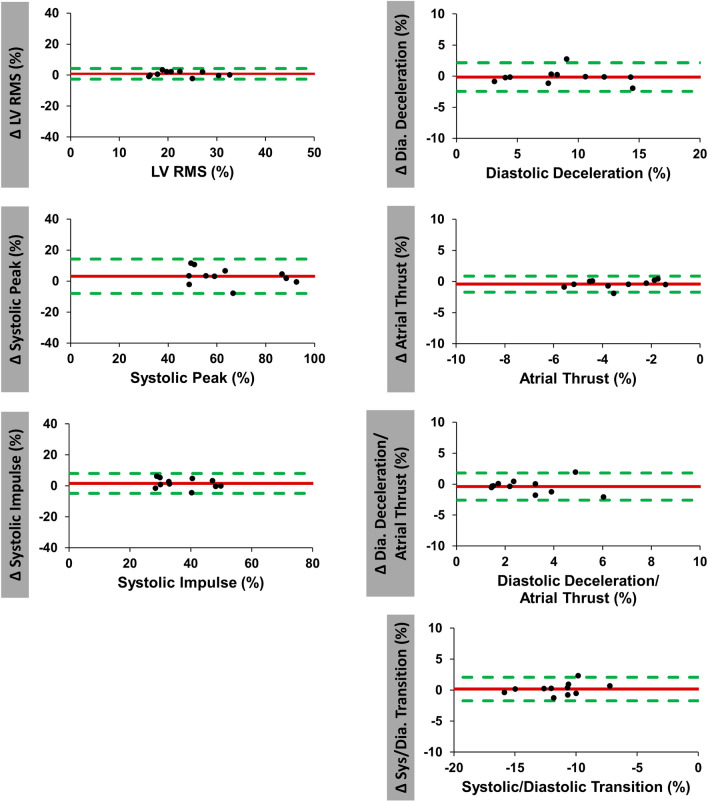
Inter-observer agreement of hemodynamic force analyses. Bland Altman plots with limits of agreement (95% confidence intervals) showing inter-observer reproducibility of hemodynamic force (HDF) analyses. Delta values (Δ) displaying the difference for inter-observer measurements. *RMS* root mean square, *LV* left ventricular.

Intra- and inter-observer reproducibility are summarized in Tables 3 and 4 as well as in Fig. 5 and 6. Both overall intra- and inter-observer reproducibility was excellent (ICC > 0.80 for all) with intra-observer assessments showing slightly lower variability compared to inter-observer analyses. Amongst intra-observer measurements LV longitudinal force showed the highest reproducibility (CoV 7.6% and ICC 0.98 [0.91–0.99]), while LV systolic/diastolic transition was most reproducible amongst inter-observer measurements (CoV 8.1% and ICC 0.97 [0.89–0.99]).

**Table 3:** Intra-observer reproducibility.

Variable	Mean difference ± SD	Coefficient of variation (%)	ICC (95% CI)
LV longitudinal force (RMS) (%)	0.8 (± 1.7)	7.6	0.98 (0.91–0.99)
Systolic peak (%)	3.1 (± 5.6)	8.6	0.97 (0.87–0.99)
Systolic impulse (%)	1.6 (± 3.2)	8.6	0.96 (0.84–0.99)
LV systolic/diastolic transition (%)	0.2 (± 1.0)	8.3	0.96 (0.87–0.99)
Diastolic deceleration (%)	0.1 (± 1.2)	13.2	0.98 (0.93–1.0)
Atrial thrust (%)	0.4 (± 0.6)	19.1	0.94 (0.72–0.98)
Diastolic deceleration/atrial thrust (%)	0.4 (± 0.4)	37.7	0.87 (0.54–0.96)

*CI* confidence interval, *ICC* intraclass correlation coefficient, *LV* left ventricular, *RMS* root mean square, *SD* standard deviation.

**Table 4:** Inter-observer reproducibility.

Variable	Mean difference ± SD	Coefficient of variation	ICC (95% CI)
LV longitudinal force (RMS)	3.0 (± 1.8)	8.5%	0.9 (0.1–0.98)
Systolic peak	9.6 (± 6.4)	10.5%	0.87 (0.1–0.97)
Systolic impulse	5.8 (± 3.6)	10.2%	0.81 (0.2–0.96)
LV systolic/diastolic transition	0.3 (± 0.9)	8.1%	0.97 (0.89–0.99)
Diastolic deceleration	0.2 (± 1.8)	20.9%	0.94 (0.79–0.99)
Atrial thrust	0.3 (± 0.6)	17.2%	0.95 (0.83–0.99)
Diastolic deceleration/atrial thrust	0.2 (± 0.9)	33.6%	0.86 (0.51–0.96)

HDF values in %.

*CI* confidence interval, *ICC* intraclass correlation coefficient, *LV* left ventricular, *RMS* root mean square, *SD* standard deviation.

### Sample size calculations

Sample sizes required for the detection of 10%, 15% or 20% relative changes in follow-up studies differed between HDF parameters (Table 5). While relatively fewer subjects allow sufficient detection of changes for systolic HDF parameters (ranging from n = 12 for the detection of a 20% relative change in systolic impulse to n = 53 for the detection of 10% relative change in LV longitudinal force) comparatively more subjects were required for diastolic parameters (ranging from n = 26 for the detection of a 20% relative change in diastolic deceleration to n = 200 for the detection of a 10% relative change in atrial thrust.

**Table 5:** Sample sizes.

Variable	Sample size 10% change	Sample size 15% change	Sample size 20% change
LV longitudinal force (RMS)	53	23	13
Systolic peak	50	23	13
Systolic impulse	49	22	12
LV systolic/diastolic transition	97	43	24
Diastolic deceleration	103	46	26
Atrial thrust	200	89	50
Diastolic deceleration/atrial thrust	141	63	35
LV global longitudinal strain (GLS)	22	10	6
LV ejection fraction (LVEF)	2	1	1

*GLS* global longitudinal strain, *LV* left ventricular, *LVEF* LV ejection fraction, *RMS* root mean square.

Established imaging parameters LVEF and LV GLS required lower sample sizes (LVEF = 2, LV GLS = 22) (Table 5).

## Discussion

This study aimed to assess the inter-study reproducibility of novel CMR-based HDF analyses derived from CMR cine images and has several notable findings. Firstly, the overall inter-study reproducibility of HDF parameters between repeated exams was sufficient with systolic HDF parameters having lower inter-study variation than diastolic HDF measurements. Secondly, smaller required sample sizes for the detection of relative changes of HDF parameters were demonstrated for systolic HDF parameters. Thirdly, overall intra- and inter-observer reproducibility for all HDF measures was excellent based on absolute agreement.

Specific blood motion patterns inside the ventricular chamber are responsible for flow-mediated forces that can be assessed by HDF analyses and were shown to be altered in failing myocardial performance^24^. Since the usage of three-dimensional/ three directional phase contrast based 4D flow techniques requires time-consuming procedures for both acquisition and post-processing^25^, advances in non-invasive CMR image post-processing software enable assessments of HDF from the motion of the LV endocardial boundary in routinely acquired cine images without the need of complex and time-consuming 4D blood flow measurements^10^.

However, at present CMR-FT-based HDF analyses are scarcely used in clinical routine and inter-study assessments for these novel parameters have not been reported yet. In our study, besides an overall sufficient inter-study reproducibility according to ICC, systolic HDF parameters showed lower inter-study variability than diastolic analyses. Similarly, required sample sizes varied between the respective HDF values and smaller sample sizes to detect relative changes of HDF measurements were observed for systolic HDF parameters. On the one hand, the relatively small study sample of the current work needs to be considered limiting a more precise detection especially of smaller absolute values and subsequently would also not allow a more precise detection of for example a 5% relative change^13^. On the other hand, required sample sizes to detect changes of other conventional imaging parameters (e.g. LVEF: 2 or LV GLS: 22 for a 10% relative change) were in line with preceding larger studies and therefore reported data can be supposed to reflect valid sample size results of HDF measurements as well^22,26^. Furthermore, previous studies assessing inter-study reproducibility of CMR-derived myocardial dyssynchrony, torsion, atrial or segmental strain on the basis of similar study participant numbers documented even larger required sample sizes (> 100) to detect similar changes for these parameters^13,27–29^. Consequently, the required sample sizes for HDF analyses can be considered within acceptable limits amongst deployed CMR parameters.

In general, comprehensive assessment and knowledge of inter-study reproducibility is a key element for successful implementation and application of a novel technique in clinical routine. Especially for the detection and monitoring of cardiovascular diseases, serial CMR examinations and subsequent post-processing rely on the assumption that changes of the imaging parameters are reliably detectable^30^.

Recently, first studies have demonstrated HDF analyses to unmask subtle impaired early diastolic filling in HFpEF patients and identifying those at an earlier stage of the disease cascade^7^. In this context, HDF analyses have been proven to outperform conventional CMR-based volumetric and deformation analyses for the detection of both systolic and diastolic impairment^11^.

Thus, HDF analyses could enable precise non-invasive monitoring of declining function in these patients. Furthermore, impaired HDF values were previously demonstrated to be associated with adverse LV remodelling after acute myocardial infarction and, moreover, HDF measurements were even suggested as potential early predictors of adverse outcome in cardiovascular diseases^31^. Consequently, CMR-based non-invasive HDF analyses possess the potential to become a clinically useful and important imaging technique for monitoring disease progression, potential treatment efficacy or deciding on the timing of therapy underlining the importance of sufficient reproducibility assessment. However, it must be noted that at present the inter-study reproducibility of HDF measurements was demonstrated to be lower compared to most commonly used LV strain assessments^13,16,29^. In this context there are several potential explanations that need to be considered when analysing HDF for deformation analyses. Since HDF assessments are based on CMR-FT measurements, they are likely to depend on known similar variability and limitations (e.g., through- or out-of-plane displacements of myocardial borders) of this technique^32^. However, the values of LV GLS did not significantly differ between both exams in our study while at least systolic HDF measurements did, indicating higher robustness of improved conventional strain analyses over just recently developed HDF assessments. In fact, the reproducibility of CMR-based strain analyses has considerably improved since their introduction and first clinical applications^13^. Similar advances might be anticipated for HDF analyses both by future software refinements and algorithm updates. Compared with strain the analyses of HDF parameters require measurements of mitral and aortic valve diameters implying an additional source of variability. This is particularly true because even minimal variations of anatomical levels occurring during image acquisition have significant impact on valve orifice measurements and subsequent HDF calculations. In this context, automated or preconfigured valve size calculation might also lead to improvements of reproducibility in the future.

Of note, while the current cine image-based HDF measurement approach requires a complete image set of 2-, 3- and 4-chamber LAX orientations, strain analyses are even possible in single image orientations and consequently allow regional assessments as compared to global HDF analyses^33^.

Besides the assessment of reproducibility, the current data suggest potential sample size adjustments, that need to be considered when applying different HDF parameters in clinical routine or studies with repeated examinations. The individual clinical usefulness of the different systolic or diastolic HDF parameters will need to be addressed in future clinical studies.

Interestingly, some discrepancies for 4D flow and cine image-derived HDF parameters detecting heart failure have been described^34^. In healthy hearts longitudinal shortening predominantly contributes to ventricular contraction, which is attenuated in heart failure or acute myocardial injury. While the 4D flow method for HDF quantification is more unlikely to be affected by decreased longitudinal contraction (at an early stage), it is possible that the endocardial dynamics-based model is more sensitive to such alterations^25^. On the other hand, the cine image-based approach might apply better in regular ventricular geometry whereas a more complex myocardial anatomy or pathological alterations could limit its applicability^8^. Considering these potential advantages and disadvantages of 4D flow and cine image-derived HDF estimations, their accuracy, comparability and interchangeability (in different cardiac diseases and altered loading conditions) have to be investigated by future studies.

Beyond the evaluation of the technique´s accuracy the current findings are also relevant for the application of these imaging biomarkers in clinical trials since higher reproducibility and increased reliability of an imaging technique could result in potentially higher cost-efficiency due to required smaller study populations^35^. In this context it is important to note, that the conditions of healthy subjects (e.g. stable lower heart rate or good scan compliance) might differ in patients and consequently results of the current work might not be fully transferrable to other (patient) cohorts. Furthermore, it is interesting to speculate whether a transfer and application of the mathematical models^10^ used by the software for CMR-FT-based HDF assessment to other myocardial strain evaluation techniques like tagging or strain-encoded (SENC) deformation imaging (both with even more challenging valve orifice assessments) would be feasible providing similar reproducibility for HDF parameters. Furthermore, the influence of CMR field strength and/or temporal as well as spatial resolution might be addressed by future studies^36,37^.

Beyond future technical refinements potentially improving reproducibility, further enhanced software applicability might be attained by fully automated HDF analyses. Artificial intelligence-based CMR-FT strain and volumetric analyses have been already shown to be feasible and to possess equally high accuracy for risk prediction compared to manual approaches^38,39^. In addition, implementation of HDF parameters beyond CMR-FT-derived strain values in novel risk prediction models might enable more precise myocardial shape and contraction analyses as well as improved risk stratification^40,41^.

Further and larger studies are needed to validate these findings and to enhance clinical applicability as well as utility of novel CMR-based HDF analyses.

### Study limitations

The main limitation of this study is its small sample size and that derived conclusions are based on the inclusion of healthy volunteers rather than patients. Consequently, it will be interesting to evaluate whether the results can be extrapolated to patients with (distinct) myocardial dysfunction. However, it is common and important to assess novel post-processing tools in healthy volunteers at first before extending the application on various cardiovascular diseases. Only in this way it is possible to understand the new image analysis method and to provide a solid basis for further technique improvement and subsequent studies including different patient collectives.

## Conclusion

CMR-FT-based non-invasive HDF analyses possess an overall good inter-study reproducibility with systolic HDF measurements showing lower inter-study variability than diastolic HDF parameters. Accordingly, at present smaller study sample sizes are required to detect relative changes for systolic HDF values compared to diastolic HDF metrics. Inter-study variability might benefit from further software refinements and future validation studies are required to enable a widespread and unrestricted clinical application of CMR-based HDF analyses.

## Data Availability

The datasets generated and analysed during the current study are not publicly available due to data protection regulations but are available from the corresponding author on reasonable request.

## Abbreviations

CI: Confidence interval
CoV: Coefficient of variation
CV: Chamber view
EDV: Enddiastolic volume
EF: Ejection fraction
ESV: Endsystolic volume
FT: Feature-tracking
GLS: Global longitudinal strain
HDF: Hemodynamic force
ICC: Intraclass correlation coefficient
LAX: Long-axis
LV: Left ventricular
CMR: Cardiovascular magnetic resonance
RMS: Root mean square
SAX: Short-axis
SV: Stroke volume
